# Genome-wide selection signatures detection in Shanghai Holstein cattle population identified genes related to adaption, health and reproduction traits

**DOI:** 10.1186/s12864-021-08042-x

**Published:** 2021-10-15

**Authors:** Dengying Liu, Zhenliang Chen, Wei Zhao, Longyu Guo, Hao Sun, Kai Zhu, Guanglei Liu, Xiuping Shen, Xiaoduo Zhao, Qishan Wang, Peipei Ma, Yuchun Pan

**Affiliations:** 1 Department of Animal Science, School of Agriculture and Biology, Shanghai Jiao Tong University, 200240 Shanghai, PR China; 2 Shanghai Dairy Cattle Breeding Centre Co., Ltd, 201901 Shanghai, P.R. China; 3 Shanghai Agricultural Development Promotion Center, 200335 Shanghai, PR China; 4 Department of Animal Breeding and Reproduction, College of Animal Science, Zhejiang University, 310058 Hangzhou, PR China

**Keywords:** Shanghai Holstein cattle population, Selection signature, Runs of homozygosity, cGTEx, Adaptation

## Abstract

**Background:**

Over several decades, a wide range of natural and artificial selection events in response to subtropical environments, intensive pasture and intensive feedlot systems have greatly changed the customary behaviour, appearance, and important economic traits of Shanghai Holstein cattle. In particular, the longevity of the Shanghai Holstein cattle population is generally short, approximately the 2nd to 3rd lactation. In this study, two complementary approaches, integrated haplotype score (iHS) and runs of homozygosity (ROH), were applied for the detection of selection signatures within the genome using genotyping by genome-reduced sequence data from 1092 cows.

**Results:**

In total, 101 significant iHS genomic regions containing selection signatures encompassing a total of 256 candidate genes were detected. There were 27 significant |iHS| genomic regions with a mean |iHS| score > 2. The average number of ROH per individual was 42.15 ± 25.47, with an average size of 2.95 Mb. The length of 78 % of the detected ROH was within the range of 1–2 MB and 2–4 MB, and 99 % were shorter than 8 Mb. A total of 168 genes were detected in 18 ROH islands (top 1 %) across 16 autosomes, in which each SNP showed a percentage of occurrence > 30 %. There were 160 and 167 genes associated with the 52 candidate regions within health-related QTL intervals and 59 candidate regions within reproduction-related QTL intervals, respectively. Annotation of the regions harbouring clustered |iHS| signals and candidate regions for ROH revealed a panel of interesting candidate genes associated with adaptation and economic traits, such as *IL22RA1, CALHM3, ITGA9, NDUFB3, RGS3, SOD2, SNRPA1, ST3GAL4, ALAD, EXOSC10*, and *MASP2*. In a further step, a total of 1472 SNPs in 256 genes were matched with 352 *cis*-eQTLs in 21 tissues and 27 *trans*-eQTLs in 6 tissues. For SNPs located in candidate regions for ROH, a total of 108 *cis*-eQTLs in 13 tissues and 4 *trans*-eQTLs were found for 1092 SNPs. Eighty-one eGenes were significantly expressed in at least one tissue relevant to a trait (*P* value < 0.05) and matched the 256 genes detected by iHS. For the 168 significant genes detected by ROH, 47 gene-tissue pairs were significantly associated with at least one of the 37 traits.

**Conclusions:**

We provide a comprehensive overview of selection signatures in Shanghai Holstein cattle genomes by combining iHS and ROH. Our study provides a list of genes associated with immunity, reproduction and adaptation. For functional annotation, the cGTEx resource was used to interpret SNP-trait associations. The results may facilitate the identification of genes relevant to important economic traits and can help us better understand the biological processes and mechanisms affected by strong ongoing natural or artificial selection in livestock populations.

**Supplementary Information:**

The online version contains supplementary material available at 10.1186/s12864-021-08042-x.

## Background

There are more than 85,000 cows and nearly 200 proven bulls in the Shanghai Holstein cattle population, which not only guarantees the basic needs of milk in Shanghai but also provides more than 3 million doses of sperm per year nationwide. Shanghai Province has long taken the leading position and become one of the largest breeding centres in China.

Holstein cattle have been extensively imported to China, mostly from Canada, the USA, France and northern Europe, since the 1940 s for use in crossbreeding aimed at improving the productivity of Chinese native cattle by combining the environmental adaptation features of the Chinese cattle with the high milk yield potential of foreign cattle [1]. The Holstein breed is categorized as a heat-sensitive cattle breed, and the most appropriate temperature for production is 5 ~ 20 degrees Celsius [2]. However, the Shanghai Holstein cattle population is exposed to long, hot and humid summers with abundant rain and temperatures of over 35 degrees Celsius. It is worth noting that Mao et al. (2015) found that the longevity of the Shanghai Holstein cattle population is generally short, approximately the 2nd to 3rd lactation [3], whereas the maximum milk yield is obtained in the fourth lactation in most Holstein populations [4].

Over several decades, a wide range of natural selection and artificial selection events in response to subtropical environments and intensive pasture and feedlot systems have greatly changed the customary behaviour, appearance, and important economic traits of Shanghai Holstein cattle. According to the theory of population genetics, the functional genes subject to selection will reveal characteristic patterns due to selection preference, and these patterns are known as “selective signatures” [5].

Recently, with the development and the prevalent application of high-throughput and cost-effective genotyping techniques, the power of detecting selection signatures at the molecular genetic level has experienced a major breakthrough. Compared to high-throughput single nucleotide polymorphism (SNP) chip technology, which has made it possible to uncover traces of positive selection and detect candidate genes based on linkage disequilibrium, next-generation sequencing (NGS) has been widely adopted by several platforms and has decreased the cost of DNA sequencing, which allows the systematic identification of selection signatures at a higher effective resolution and sensitivity [5]. Furthermore, studies based on sequence data do not suffer from SNP ascertainment bias, as do studies that are performed using commercially available SNP assays [6, 7].

To date, various analytical methods have been proposed to detect different kinds of selection signatures. A considerable amount of research has been conducted to detect selection signatures in selected populations during the last decade by using various statistical approaches, including Tajima’s D [8], extended haplotype homozygosity (EHH) statistic [9], integrated Haplotype Score (iHS) [10], cross-population Extended Haplotype Homozygosity (XPEHH) and cross-population composite likelihood ratio (XP-CLR) [11]. EHH is a popular approach that is known for reliably detecting ongoing selection and is a long haplotype-based test. In populations under positive selection, the mutation frequency will rapidly increase. Therefore, regions with extremely strong and long-range LD with high haplotype allele frequencies can be used to detect recent selection by EHH [12]. The iHS approach was developed by Voight et al. (2006); it is based on EHH and can overcome the influence of heterogeneous recombination rates across the genome. iHS can distinguish the ancestral and derived alleles of a polymorphic site, as a much larger EHH score for the derived allele than for the ancestral allele is expected to represent positive selection. iHS is usually sensitive for detecting positive selection signatures for intermediate frequency variants [13].

Signatures of selection could be observed in genome-wide ROH scans in animals. It was suggested that genetic diversity is reduced under selective pressure; thus, ancestral genetic variations are often transformed into long stretches of consecutive homozygous genotypes across the genome [14]. The size and frequency of ROH vary according to population diversity and selection pressure. Analyses of ROH allow the identification of genomic regions with possible selection signatures for breed [15]. There are many studies with ROH for the identification of selection signatures in cattle [16, 17], sheep [18, 19], horses [20, 21] and pigs [22, 23]. To improve the power and spatial resolution for identifying selection signatures, combining multiple methods into composite tests is useful for mapping the comprehensive footprint of selection across the genome.

Genome-scale bioinformatics annotations are available from a number of sources, including the cattle Genotype-Tissue Expression (cGTEx, http://cgtex.roslin.ed.ac.uk/) atlas, which represents the most comprehensive reference resource of the cattle transcriptome to date and is based on 11,642 RNA sequences from publicly available datasets representing over 100 cattle tissues. This database provides a detailed characterization of the genetic control of gene regulation across 24 major tissues and provides novel biological insights into the molecular regulatory mechanisms underpinning agronomic traits in cattle by conducting a transcriptome-wide association study (TWAS) linking gene expression in different tissues with 43 economically important traits [24]. Mapping expression quantitative trait loci (eQTLs) has provided great supporting evidence for important insights into the regulatory pathways involved in disease, which contain genetic variants associated with gene expression. We defined genes whose expression levels were significantly associated with SNPs as eGenes. eGenes in a disease-relevant tissue provide insight into gene regulation and are genes whose expression levels are associated with genetic variants. cGTEx data were used to estimate the variation in gene expression regulation between tissues by comparison with our data. If the selective signatures or the candidate genes identified are also eQTLs or eGenes, then there is strong evidence to further study these variants or genes.

However, little information is available from the scientific literature about the selection signatures and population structure of the Shanghai Holstein population. Therefore, the aims of this study were to 1, investigate the recent selective signatures based on next-generation sequencing data in the Shanghai Holstein population in response to local climatic conditions and artificial selection for economical purposes, and 2, explore the genes related to biological processes and traits of interest to identify important functional candidate genes undergoing positive selection in Shanghai Holstein cattle.

## Results

After filtration, we utilized 164,312 high-quality SNPs for analysis of the genomic selection signature in our study. These SNPs covered 2.51 Gb of the cattle genome (UMD3.1), with an average distance of 15.56 kb between adjacent SNPs. The average distance between the adjacent SNPs across autosomes ranged from 7.43 kb on BTA25 to 22.49 kb on BTA6, and the standard deviation of the adjacent SNPs ranged from 18.86 on BTA25 to 46.70 on BTA1, which is presented in Additional file 1: Table S1. An overview of the relationships among these animals from 24 farms is presented in Additional file 1: Figure S1. We further estimated the inbreeding coefficient based on the genomic information for all Shanghai Holstein cattle. The average inbreeding coefficients estimated using GCTA (-ibc command) are shown in Additional file 1: Table S2. The inbreeding coefficient reflects the deviations in the observed inbreeding from the expected values in the current population, and the average inbreeding coefficient of this population was 0.36.

### Detection of selection signature using iHS approach

**Fig. 1:**
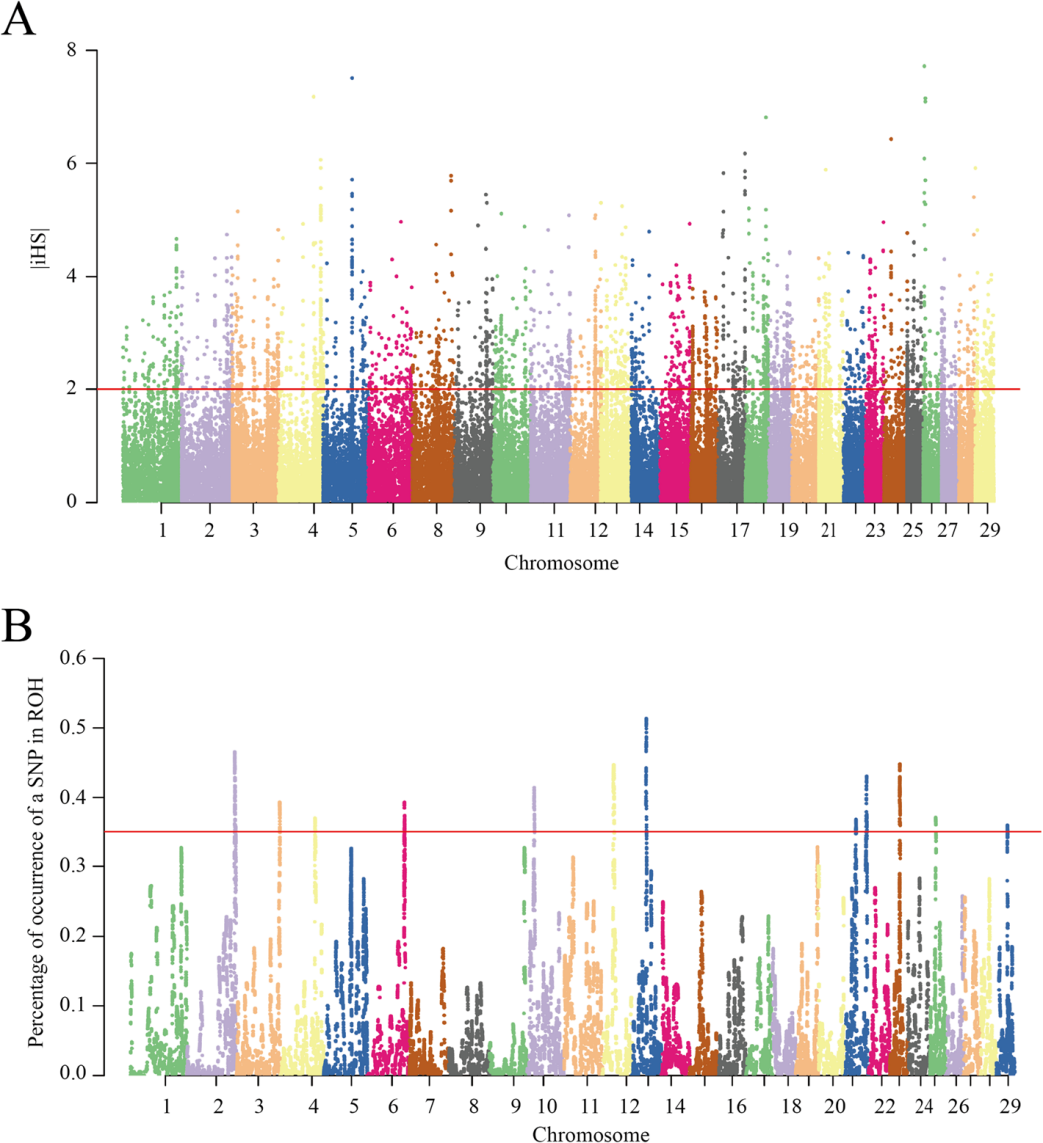
**A** Genome-wide distribution of selection signatures detected by iHS. The dashed line represents the threshold levels of top 1 % (|iHS| = 2); **B** Manhattan plot of incidence of each SNP in the ROH across individuals. The dashed line represents the top 1 % threshold

The iHS test was used to detect strong footprints of recent selection within the Shanghai Holstein cattle population. We obtained a total of 62,140 SNPs with estimated |iHS| scores (Additional file 2). The chromosome-wide scans of iHS for the studied population are shown in Fig. 1A. The plots show clear evidence of selective forces in different regions of the genome. A total of 9304 regions were detected based on the single site |iHS| score. Based on the top 1 % of values, 101 candidate regions were identified. In total, 256 candidate genes overlapped with these significant iHS genomic regions according to the UMD 3.1 assembly (Additional file 3). Among the 101 candidate regions, there were 27 significant |iHS| genomic regions with a mean |iHS| score > 2, and 84 genes were located in these regions including *ITGA9, MASP2, SLC39A11, SSTR2, TGFBR1, TSPEAR, LRRC3, SNRPA1, PFKL, RGS3, SRSF10, SUPT3H, FUCA1, CSPP1, IL22RA1*, and *AOX2*. (Table 1). The region located on BTA6 (BTA6:78,000,000–78,500,000) had the highest |iHS| score (2.942), and no candidate gene overlapped with this region.

**Table 1:** Genomic region and associated genes of the top 27 significant |iHS|

Chr	Start (bp)	End (bp)	Mean |iHS| Value	No. of SNP	Candidate Genes
6	78,000,000	78,500,000	2.94	2	—
13	34,000,000	34,500,000	2.88	1	SVIL、ZNF438
2	92,500,000	93,000,000	2.68	18	AOX1、AOX2、AOX4、BZW1、CLK1、NIF3L1、ORC2、PPIL3、SGO2
13	40,000,000	40,500,000	2.67	9	KIZ
21	28,750,000	29,250,000	2.66	21	SNRPA1、TARS3、TM2D3
14	30,750,000	31,250,000	2.60	5	ARFGEF1、COPS5、CSPP1、PPP1R42、SGK3
24	20,000,000	20,500,000	2.55	27	CELF4
21	29,000,000	29,500,000	2.53	23	SNRPA1
2	134,250,000	134,750,000	2.37	1	CNR2、FUCA1、IFNLR1、IL22RA1、PNRC2、SRSF10
24	19,750,000	20,250,000	2.35	29	CELF4
5	79,000,000	79,500,000	2.29	89	RBFOX2、LOC515697
26	2,750,000	3,250,000	2.29	38	—
2	92,750,000	93,250,000	2.27	22	AOX2、AOX4、BZW1、CFLAR、CLK1、NDUFB3、NIF3L1、ORC2、PPIL3
16	39,250,000	39,750,000	2.24	13	EXOSC10、MASP2、SRM、TARDBP
22	11,000,000	11,500,000	2.24	9	CTDSPL、ITGA9、MIR2367、MIR26A-1、MIR26C、VILL
26	3,000,000	3,500,000	2.24	39	—
8	107,500,000	108,000,000	2.22	13	ALAD、BSPRY、C8H9orf43、CDC26、HDHD3、POLE3、PRPF4、RGS3、RNF183、SLC31A1、SLC31A2
1	113,500,000	114,000,000	2.20	23	GMPS、SLC33A1
23	28,000,000	28,500,000	2.18	8	MIR12033、OR12D2、UBD
1	147,000,000	147,500,000	2.15	34	CFAP410、TSPEAR、FAM207A、ITGB2、LRRC3、PFKL、PTTG1IP、UBE2G2、SUMO3、KRTAP10-2、KRTAP10-8、KRTAP12-2、LOC617218、LOC780781
8	66,500,000	67,000,000	2.14	9	ALG2、COL15A1、SEC61B、TGFBR1
27	1,000,000	1,500,000	2.10	16	CLN8、MYOM2、MIR10169
6	78,250,000	78,750,000	2.08	4	—
19	59,750,000	60,250,000	2.06	7	SLC39A11、SSTR2
14	58,500,000	59,000,000	2.03	1	—
23	19,000,000	19,500,000	2.02	16	SUPT3H
18	10,500,000	11,000,000	2.00	13	COX4I1、EMC8、IRF8

### Genomic distribution of runs of homozygosity

**Fig. 2:**
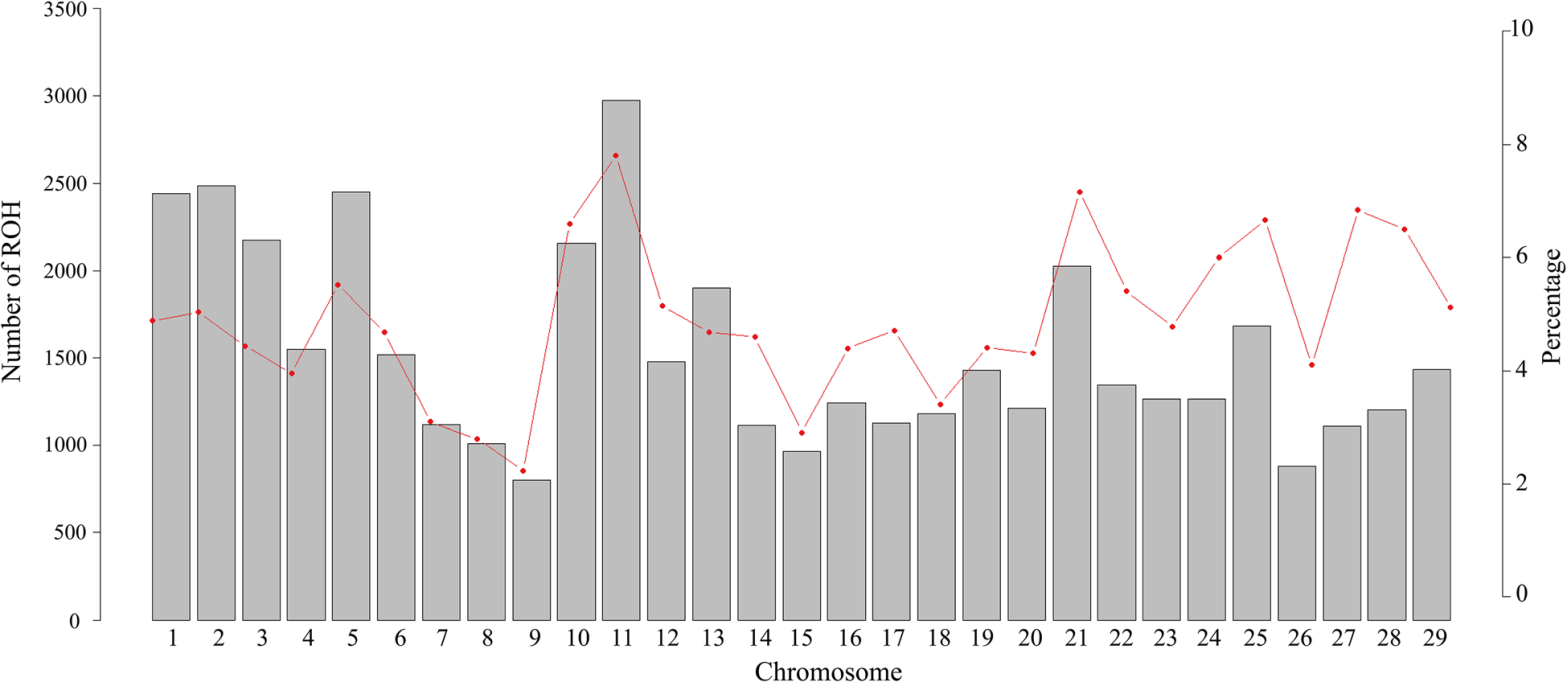
Number of ROH longer than 1 Mb per chromosome (bars) and average percentage of each chromosome covered by ROH (red line)

In total, 44,509 ROH among 1092 samples were identified. The average number of ROH per individual was 42.15 ± 25.47, ranging from 1 to 121 ROH. The genomic distribution of ROH was nonuniform both in length and position across chromosomes. The average length of ROH was 2.95 Mb across all autosomes, but the total length of ROH per individual varied considerably from 1.51 Mb to 430 Mb. The longest segment was 18.82 Mb in length (381 SNPs) and was found on BTA6. Figure 2 shows the percentage of bovine chromosomes covered by ROH and the highest coverage by ROH was observed on BTA11 (7.8 % of chromosomal length), whereas the lowest was on BTA9 (2.24 % of chromosomal length). The number of ROH per chromosome was greatest for BTA11 (2976 segments) and lowest for BTA9 (800 segments) (Fig. 2).

In this study, we classified ROH into four different categories according to their physical length: 1 to < 2 Mb, 2 to < 4 Mb, 4 to < 8 Mb and > 8 Mb. Descriptive statistics of each length category are given in Table 2. Our results show that 99 % of the ROH were shorter than 8 Mb. The total length of ROH for the Shanghai Holstein cattle population was composed mostly of shorter segments (1–2 Mb and 2–4 Mb). These segments accounted for approximately 78 % of all ROH detected, which contributed to 0.79 % and 2.16 % of the cumulative length of ROH. ROH (1–2 Mb) were more abundant throughout the genome than ROH (4–8 Mb); however, the proportion of the genome covered by ROH (1–2 Mb) was much smaller than that covered by ROH (4–8 Mb).

**Table 2:** Descriptive statistics of runs of homozygosity (ROH) number and length (in Mb) by ROH length class (ROH 1–2 Mb, ROH 2–4 Mb, ROH 4–8 Mb, ROH > 8 Mb and total)

Class	No. of ROH	Percent (%)	Mean length	Standard deviation	Genome coverage (%)
ROH_1 − 2 Mb_	13,992	31.00	1.54	0.28	0.79
ROH_2 − 4 Mb_	20,915	47.00	2.84	0.57	2.16
ROH_4 − 8 Mb_	9279	21.00	5.08	0.89	1.72
ROH_> 8 Mb_	323	1.00	9.41	1.37	0.11
Total	44,509	100	2.95	1.49	4.8

To identify the genomic regions that were most commonly associated with ROH in all individuals, the top 1 % of SNPs observed in the ROH was selected, and the adjacent SNPs over this threshold were merged into genomic regions corresponding to ROH islands [25]. In the ROH islands detected here, each SNP showed a percentage of occurrence > 30 % (occurring in over 30 % of the samples) (Fig. 1B). This approach resulted in the identification of 18 ROH islands across 16 autosomes, with one on BTA1, 3, 4, 6, 9, 10, 11, 12, 13, 19, 20, 23, 25 and 29 and two on BTA2 and 21, and the length of these regions ranged from 679 kb on BTA11 to 2.79 Mb on BTA12 (Table 3). Among the described ROH islands, the strongest pattern was observed on BTA13:39,852,457–41,196,648, with an overlapping ROH region present in 50 % of the samples.

**Table 3:** List of genomic regions of extended homozygosity detected in Shanghai Holstein cattle population

Chr	Start (bp)	End (bp)	Length (bp)		SNPs	No. of Genes		Candidate Genes
13	39,852,457	41,196,648	1,344,191	120		3	KIZ、NKX2-2、XRN2	
2	135,983,904	137,968,708	1,984,804	134		17	ALPL、CAMK2N1、CDA、CDC42、CELA3B、DDOST、ECE1、HP1BP3、KIF17、LOC515042、LOC789612、MIR2284U、MUL1、PINK1、RAP1GAP、SH2D5、ZBTB40	
12	30,773,910	33,562,640	2,788,730	117		21	ATP8A2、CDX2、FLT1、GPR12、GSX1、GTF3A、LNX2、MIR2285O-2、MIR2300A、MIR2300B、MTIF3、PDX1、POLR1D、POMP、RNF6、RPL21、SHISA2、SLC46A3、URAD、USP12、WASF3	
21	31,435,280	33,563,201	2,127,921	81		17	COMMD4、CSPG4、ETFA、HMG20A、IMP3、ISL2、MAN2C1、MIR631、NEIL1、ODF3L1、PSTPIP1、PTPN9、RCN2、SNUPN、SNX33、TMEM266、TSPAN3	
10	18,844,833	21,194,474	2,349,641	98		43	ADCY4、ADPGK、ARIH1、BBS4、CBLN3、CELF6、CHMP4A、CIDEB、DCAF11、DHRS1、FITM1、GMPR2、HEXA、INSYN1、IPO4、IRF9、KHNYN、LTB4R、LTB4R2、MDP1、MIR11989、MIR12031、NEDD8、NFATC4、NOP9、NPTN、NR2E3、NYNRIN、PARP6、PKM、PSME1、PSME2、RABGGTA、REC8、RIPK3、RNF31、SDR39U1、TBC1D21、TGM1、THSD4、TINF2、TM9SF1、TSSK4	
23	27,948,371	29,204,475	1,256,104	85		4	MIR12033、OR12D2、TRIM27、UBD	
3	123,888,189	124,870,683	982,494	92		10	ASB1、ESPNL、HES6、ILKAP、MIR2902、PER2、SCLY、TRAF3IP1、TWIST2、UBE2F	
4	98,248,048	100,380,507	2,132,459	95		5	CHCHD3、EXOC4、MIR2423、MIR320B、PLXNA4	
25	15,526,462	16,728,810	1,202,348	91		2	ABCC1、ABCC6	
6	107,592,661	108,740,633	1,147,972	58		3	EVC、EVC2、MSX1	
21	61,326,146	64,094,599	2,768,453	103		6	AK7、ATG2B、BDKRB1、GSKIP、PAPOLA、VRK1	
29	30,857,817	31,924,963	1,067,146	105		8	DCPS、FAM118B、FOXRED1、KIRREL3、RPUSD4、SRPRA、ST3GAL4、TIRAP	
1	144,773,205	145,465,387	692,182	49		4	C2CD2、RIPK4、TMPRSS2、ZBTB21	
9	99,670,895	101,121,625	1,450,730	85		10	ACAT2、AGPAT4、AIRN、IGF2R、MRPL18、PLG、SLC22A1、SOD2、TCP1、WTAP	
19	62,790,612	63,501,716	711,104	48		8	ABCA9、AMZ2、ARSG、MGC134105、PRKAR1A、RGS9、SLC16A6、WIPI1	
11	25,226,313	25,905,694	679,381	29		2	PKDCC、MIR12030	
20	780,499	2,012,217	1,231,718	70		5	FOXI1、KCNMB1、LCP2、SLIT3、SPDL1	
2	139,926,793	140,719,836	793,043	69		0	——	

Within all of the ROH islands reported here, a reasonable number of genes (n = 168) was observed (Table 3). We found that some SNPs in ROHs were located in intergenic regions, and a few genes were detected in some identified regions. For example, the length of region BTA25:15,526,462–16,728,810 was 1.2 Mb, but only contained 2 annotated genes. Although the length of the region on BTA2 was 793 kb, no gene was detected. The possible reasons were either that the annotation of the cow reference genome is still incomplete or that the genomic region was positioned in a noncoding region.

### Overlap between selection metrics

In total, 101 and 18 candidate regions were detected by the iHS approach and ROH estimation, respectively. All the candidate regions were spread across 27 of the 29 autosomal chromosomes of the bovine genome. Coincident signatures identified by both methods were found on chromosomes BTA12, BTA13, BTA21 and BTA23 (Table 4). There were 11 common genes revealed by both iHS and ROH analyses: *MTIF3, UBD, OR12D2, KIZ, LNX2, USP12, POLR1D, GTF3A, GSX1, RPL21*, and *MIR12033*. The majority of the 11 common genes detected by both methods were related to heat stress, reproduction, immunity, and mastitis.

**Table 4:** List of overlapped genomic regions detected by iHS and ROH

iHS candidate regions					ROH candidate regions							Genes in regions
**Chr**	**Start (bp)**	**End (bp)**	**Mean |iHS| Value**			**Chr**		**Start (bp)**	**End (bp)**	**Length**	**SNPs**	
12	32,000,000	32,500,000		1.65	12		30,773,910		33,562,640	2,788,730	117	MTIF3、LNX2、USP12、POLR1D、GTF3A、GSX1、RPL21
13	40,000,000	40,500,000		2.67	13		39,852,457		41,196,648	1,344,191	120	KIZ
21	63,000,000	63,500,000		2.00	21		61,326,146		64,094,599	2,768,453	103	——
23	28,000,000	28,500,000		2.18	23		27,948,371		29,204,475	1,256,104	85	MIR12033、OR12D2、UBD

### Functional analyses of the candidate genes

A total of 256 and 168 genes were fully or partially contained within each selected region, as detected by iHS and ROH, and were subjected to GO annotation and KEGG pathway enrichment to further analyze their biological functions. Multiple categories were statistically significant (*P* value < 0.05).

All the genes from the iHS method were grouped into several different annotation clusters, of which 18 GO terms and 5 KEGG pathways were significantly enriched (Additional file 1: Table S3). The enriched annotation terms from iHS analysis were associated with different molecular functions, biological processes, and cellular components. A gene list analysis revealed a high percentage of genes involved in intracellular part (GO:0044424), organic substance metabolic process (GO:0071704), cellular metabolic process (GO:0044237), primary metabolic process (GO:0044238), nitrogen compound metabolic process (GO:0006807) and membrane-bounded organelle (GO:0043227) and organelle part (GO:0044422). Similarly, the 5 KEGG pathways included vitamin B6 metabolism, folate biosynthesis, tryptophan metabolism, metabolic pathways and valine, leucine and isoleucine degradation.

All the genes detected in all ROH were grouped into several different annotation clusters (Additional file 1: Table S4). 16 GO terms (2 molecular functions, 6 biological processes, and 8 cellular components) and 1 KEGG pathway were enriched. Biological terms were further labelled based on the following categories: positive regulation of sequence-specific DNA binding transcription factor activity (GO:0051091), cell growth (GO:0016049), macromolecule localization (GO:0033036), developmental growth (GO:0048589), regulation of molecular function (GO:0065009), and primary metabolic process (GO:0044238).

### QTLs based on identified regions

QTLs and selection signatures in the same location indicate a precise relationship between the selection for traits and the effects of variation at a locus [12]. Thus, we investigated the candidate regions overlapping QTL regions extracted from the cattle QTLdb. Overall, 89 genomic regions for 101 candidate regions detected by the *iHS* method overlapped with QTLs. We compared the significant regions with the QTL regions associated with health and reproduction. As shown in Additional file 4, 52 significant regions were located within the health-related QTLs, and 59 significant regions were located within the reproduction-related QTLs. The candidate regions detected in the present study contain several QTLs for important health-related traits in cattle, including somatic cell score (SCS), heat tolerance, and clinical mastitis. Twenty-six candidate regions overlapped with the somatic cell score, 7 candidate regions overlapped with clinical mastitis, and 4 candidate regions overlapped with heat tolerance. Simultaneously, 21 candidate regions overlapped with calving ease, 18 candidate regions overlapped with inseminations per conception, 15 candidate regions overlapped with stillbirth, and 15 candidate regions overlapped with interval to first oestrus after calving.

Furthermore, 160 and 167 genes were associated with the 52 and 59 candidate regions within the health-related and reproduction-related QTL intervals, respectively. The results also showed that a significant region might overlap with several QTLs associated with different traits. Among 89 candidate regions, 35.96 % (32 regions) were related to both health traits and reproductive traits. In total, 104 genes were detected in these 32 candidate regions and included 28 genes overlapping with the most significant genomic regions (mean |iHS| score > 2). These genes were *CNR2, UBD, TSPEAR, UBE2G2, SUPT3H, FUCA1, IFNLR1, IL22RA1, PNRC2, SRSF10, EXOSC10, MASP2, SRM, TARDBP, MIR12033, OR12D2, CFAP410, FAM207A, ITGB2, LRRC3, PFKL, PTTG1IP, SUMO3, KRTAP10-2, KRTAP10-8, KRTAP12-2, LOC617218* and *LOC780781*. The 28 genes were used to determine whether other literature reported the function of these genes. Sixteen genes overlapped with candidate regions with a mean |iHS| >2 and were only related to health QTLs; 23 genes overlapped with candidate regions with a mean |iHS| >2 and were only related to reproductive QTLs (Table 5). In particular, 12 genes overlapped with 4 candidate regions related to heat tolerance QTLs, *ADCK1, GOLGA4, ITGA9, ECI2, PRPF4B, PXDC1, ADIPOR2, ALKBH1, DCP1B, SLIRP, SNW1*, and *SPTLC2.*

**Table 5:** Health-related and Reproduction-related genomic region with mean |iHS| value >2 and associated genes

QTL	Chr	Start (bp)	End (bp)	Mean |iHS| Value	Genes
Health-related	2	92,500,000	93,000,000	2.68	AOX1、AOX2、AOX4、BZW1、CLK1、NIF3L1、ORC2、PPIL3、SGO2
	2	92,750,000	93,250,000	2.27	AOX2、AOX4、BZW1、CFLAR、CLK1、NDUFB3、NIF3L1、PPIL3、ORC2
	22	11,000,000	11,500,000	2.24	CTDSPL、ITGA9、MIR2367、MIR26A-1、MIR26C、VILL
	8	107,500,000	108,000,000	2.22	ALAD、BSPRY、C8H9orf43、CDC26、HDHD3、POLE3、PRPF4、RNF183、RGS3、SLC31A1、SLC31A2
	18	10,500,000	11,000,000	2.00	COX4I1、EMC8、IRF8
Reproduction-related	6	78,000,000	78,500,000	2.94	——
	13	34,000,000	34,500,000	2.88	SVIL、ZNF438
	13	40,000,000	40,500,000	2.67	KIZ
	21	28,750,000	29,250,000	2.66	SNRPA1、TARS3、TM2D3
	14	30,750,000	31,250,000	2.60	ARFGEF1、COPS5、CSPP1、PPP1R42、SGK3
	1	113,500,000	114,000,000	2.20	GMPS
	8	66,500,000	67,000,000	2.14	ALG2、COL15A1、SEC61B、TGFBR1
	27	1,000,000	1,500,000	2.10	CLN8、MIR10169、MYOM2
	6	78,250,000	78,750,000	2.08	——
	19	59,750,000	60,250,000	2.06	SLC39A11、SSTR2
	14	58,500,000	59,000,000	2.03	——
Health-related and Reproduction-related	2	134,250,000	134,750,000	2.37	CNR2、FUCA1、IFNLR1、IL22RA1、PNRC2、SRSF10
	26	2,750,000	3,250,000	2.29	——
	16	39,250,000	39,750,000	2.24	EXOSC10、MASP2、SRM、TARDBP
	23	28,000,000	28,500,000	2.18	MIR12033、OR12D2、UBD
	1	147,000,000	147,500,000	2.15	CFAP410、FAM207A、ITGB2、KRTAP10-2、KRTAP10-8、KRTAP12-2、LOC617218、LOC780781、LRRC3、PFKL、PTTG1IP、SUMO3、TSPEAR、UBE2G2
	23	19,000,000	19,500,000	2.02	SUPT3H

### Using cGTEx in multiple tissues to discover eQTLs and eGenes

We uploaded the Ensembl IDs of the 256 and 168 genes detected by the *iHS* method (the top 1 %) and ROH analysis (top 1 %), respectively, to cGTEx. All *cis*-eQTLs and *trans*-eQTLs associated with the expression of these genes were then downloaded from the website. UCSC liftOver tools were used to convert 1549 SNPs located in 256 candidate genes to ARS-UCD1.2 for consistency. A total of 1472 SNPs were matched with 352 *cis*-eQTLs in 21 tissues and 27 *trans*-eQTLs in 6 tissues based on the 256 candidate genes (Additional file 5: Table S5; Additional file 5: Table S6;). For SNPs located in candidate regions for ROH, a total of 108 *cis*-eQTLs in 13 tissues and 4 *trans*-eQTLs in blood were found among 1092 SNPs converted from 1101 SNPs (Additional file 5: Table S7; Additional file 5: Table S8). Eighty-one eGenes were significantly expressed in at least one tissue and related to at least one trait (*P* value < 0.05) by matching the 256 genes detected by iHS with the 496 gene-tissue pairs significantly associated with the 43 economically important traits from cGTEx (Table 6). Thirteen gene-tissue pairs (*KCTD18*-Adipose, *SPATS2L*-Blood, *AOX1*-Adipose, *UNC13B*-Blood, *COQ2*-Adipose, *FAM228B*-Liver, *ALDH6A1*-Liver, *STOML2*-Mammary, *GTF3A*-Embryo, *CHRND*-Intramuscular_fat, *PPP1R42*-Intramuscular_fat, *TTC22*-Blood, and *VPS35L*-Liver) were significantly associated with more than 5 traits; 7 gene-tissue pairs (*FAM135B*-uterus, *FAM228B*-liver, *CFAP410*-liver, *GTF3A*-embryo, *CHTF8*-liver, *MINDY4*-hypothalamus, and *VAPB*-macrophage) were associated with milk production traits; 5 gene-tissue pairs (*RPS6KA4*-Uterus, *INMT*- Liver, *MINDY4*-Hypothalamus, and *GGCT*-Milk_cell) were associated with productive life; *ITGB2*-Muscle, *NCEH1*-Blood and *ABCD4*-Blood were significantly associated with SCS; *TARS3*-Adipose and *USP12*-Intramuscular _fat were significantly associated with mastitis; 30 gene-tissue pairs were significantly associated with reproduction traits; *FAM228B*-Liver was associated with heifer conception rate, cow conception rate, daughter calving ease and daughter pregnancy rate; and *VPS35L*- Liver was significantly associated with sire still birth, sire calving ease, age at first calving and daughter still birth (Table 7; Additional file 5: Table S9). For the 168 significant genes detected by ROH analysis, 47 gene-tissue pairs were significantly associated with at least one of the 37 traits (Table 6). Seven gene-tissue pairs (*ETFA*-liver, *C2CD2*-embryo, *GTF3A*-embryo, *TRIM27*-macrophage, *KIRREL3*-adipose, *GSKIP*-muscle, and *FLT1*-macrophage) were significantly associated with more than 5 traits; 6 gene-tissue pairs were associated with milk production traits; *C2CD2*-embryo was associated with milk yield, protein yield, protein percentage, and fat yield; 22 gene-tissue pairs were associated with reproductive traits; and 7 gene-tissue pairs *(ETFA*-Liver, *ATP8A2*-Liver, *CIDEB*-Blood, *LTB4R*-Blood, *PTPN9*-Muscle, *MAN2C1*-Blood, and *USP12*-Intramuscular_fat) were significantly associated with mastitis (Table 8; Additional file 5: Table S10). These eQTLs and eGenes provide great supporting evidence for our genome-wide selection signatures and important insights into the regulatory pathways involved in many diseases.

**Table 6:** The number of *cis*-eQTLs, *trans*-eQTLs and gene-tissue pairs discovered by using cGTEx

Methods	Genes	SNPs	*cis*-eQTLs	tissues	*trans*-eQTLs	tissues	Gene-tissue pairs
iHS	256	1549	352	21	27	6	81
ROH	168	1101	108	13	4	1	47

**Table 7:** The gene-tissue pairs significantly associated with economically important traits from cGTEx (for iHS)

Traits	Gene-tissue pairs
Milk yield	C2CD2-Embryo、GSKIP-Muscle、SNX33-Embryo
Protein yield	C2CD2-Embryo、GSKIP-Muscle、SNX33-Embryo、KIRREL3-Adipose、HES6-Intramuscular_fat
Protein percentage	C2CD2-Embryo、GTF3A-Embryo
Fat yield	C2CD2-Embryo、KIRREL3-Adipose
Mastitis	ETFA-Liver、ATP8A2-Liver、CIDEB-Blood、LTB4R-Blood、PTPN9-Muscle、MAN2C1-Blood、USP12-Intramuscular_fat
Sire conception rate	TRIM27-Macrophage、GSKIP-Muscle、RNF31-Milk_cell、ADCY4-Ileum、ATG2B-Liver、SDR39U1-Intramuscular_fat
Sire calving ease	RGS9-Blood、ARSG-Blood
Heifer conception rate	RPUSD4-Adipose
Age at first calving	CHMP4A-Blood、DCAF11-Blood、HP1BP3-Lymph_node、WIPI1-Jejunum
Days to first breeding after calving	GTF3A-Embryo、MGC134105-Liver
Daughter stillbirth	FLT1-Macrophage、RGS9-Blood、TCP1-Blood
Cow conception rate	EXOC4-Blood、MGC134105-Liver
Daughter pregnancy rate	TRIM27-Macrophage、EXOC4-Blood、CHMP4A-Blood、MGC134105-Liver、CAMK2N1-Blood
Daughter calving ease	PRKAR1A-Blood、RGS9-Blood、CBLN3-Adipose、KCNMB1-Blood
Under depth	ETFA-Liver、FLT1-Macrophage
Udder cleft	ETFA-Liver、TRIM27-Macrophage、FLT1-Macrophage
Teat length	PTPN9-Muscle
Strength	GTF3A-Embryo、FLT1-Macrophage、KIRREL3-Adipose、PRKAR1A-Blood
Stature	GTF3A-Embryo
Rump width	KIRREL3-Adipose、ST3GAL4-Blood、ARSG-Blood、FOXRED1-Blood
Rump angle	GTF3A-Embryo、GSKIP-Muscle
Retained placenta	C2CD2-Embryo、CHMP4A-Blood、MDP1-Blood、TMPRSS2-Mammary
Rear udder height	ETFA-Liver、TRIM27-Macrophage、PAPOLA-Monocytes
Rear teat placemen	ETFA-Liver、KHNYN-Intramuscular_fat、PKM-Macrophage
Rear legs rear view	SRPRA-Blood、ALPL-Blood
Net merit	C2CD2-Embryo、TRIM27-Macrophage
Metritis	EXOC4-Blood、CIDEB-Blood、LTB4R-Blood、ABCC6-Liver
Ketosis	FLT1-Macrophage、ATP8A2-Liver、LTB4R-Blood、DCAF11-Blood、RNF31-Milk_cell、FITM1-Blood、MRPL18-Blood
Hypocalcemia	ATP8A2-Liver
Front teat placement	ETFA-Liver、CIDEB-Blood
Fore udder attachment	ETFA-Liver、GSKIP-Muscle、ST3GAL4-Blood、DCPS-Liver
Feet and legs	SRPRA-Blood
Displaced abomasum	ATP8A2-Liver、EXOC4-Blood、HP1BP3-Lymph_node
Dairy form	TRIM27-Macrophage、ACAT2-Liver
Body depth	KIRREL3-Adipose、PRKAR1A-Blood
Foot angle	GTF3A-Embryo、SRPRA-Blood
Overall conformation score	ETFA-Liver、SRPRA-Blood、ST3GAL4-Blood

**Table 8:** The gene-tissue pairs significantly associated with economically important traits from cGTEx (for ROH)

Traits	Gene-tissue pairs
Milk yield	FAM135B-Uterus
Protein yield	FAM228B-Liver、CFAP410-Liver
Protein percentage	FAM228B-Liver、CHTF8-Liver、GTF3A-Liver
Fat percent	MINDY4-Hypothalamus、VAPB-Macrophage
Productive lift	ALDH6A1-Liver、RPS6KA4-Uterus、INMT-Liver、MINDY4-Hypothalamus、GGCT-Milk_cell
Somatic cell score	ITGB2-Muscle、NCEH1-Blood、ABCD4-Blood
mastitis	TARS3-Adipose、USP12-Intramuscular_fat
Sire stillbirth	VPS35L-Liver、EIF4E2-Intramuscular_fat、PDE6D-Mammary
Sire conception rate	NOD1-Adipose
Sire calving ease	VPS35L-Liver、SLC31A1-Liver、FUCA1-Blood
Heifer conception rate	COQ2-Adipose、FAM228B-Liver、TP53I3-Adipose、FKBP1B-Oviduct、LIN54-Lung
Age at first calving	UNC13B-Blood、ALDH6A1-Liver、PPP1R42-Intramuscular_fat、VPS35L-Liver、PRG4-Liver、UBE2G2-Intramuscular_fat
Days to first breeding after calving	KCTD18-Adipose、SPATS2L-Blood、AOX1-Adipose、GTF3A-Embryo、PRPF4B-Embryo、SNRPA1-Adipose
Daughter stillbirth	UNC13B-Blood、VPS35L-Liver、ATAD2B-Skin_fibroblast、CELF4-Ileum、LRRC3-Liver、SYNDIG1L-Adipose
Cow conception rate	KCTD18-Adipose、FAM228B-Liver、TTC22-Blood、SRM-Mammary、PFKL-Intramuscular_fat
Daughter pregnancy rate	KCTD18-Adipose、SPATS2L-Blood、AOX1-Adipose、FAM228B-Liver、SRM-Mammary
Daughter calving ease	FAM228B-Liver、PPP1R42-Intramuscular_fat、RAB22A-Milk_cell
Under depth	ALDH6A1-Liver
Udder cleft	KCTD18-Adipose、SPATS2L-Blood、AOX1-Adipose、UNC13B-Blood、COQ2-Adipose、ALDH6A1-Liver、CHRND-Intramuscular_fat、TP53I3-Adipose、IQCK-Adipose、MACROD1-Liver、MOCS1-Adipose、PNRC2-Blood、HPSE-Blood、CRHR2-Jejunum、GARS1-Embryo
Teat length	UNC13B-Blood、EXOSC10-Mammary
strength	COQ2-Adipose、GTF3A-Embryo、PPP1R42-Intramuscular_fat、TTC22-Blood、ADIPOR2-Hypothalamus、FAM228A-Hypothalamus、UBXN2A-Blood、CSPP1-Blood、MOCS1-Adipose、WNT11-Macrophage
stature	STOML2-Mammary、GTF3A-Embryo、ADIPOR2-Hypothalamus、FAM228A-Hypothalamus、UBXN2A-Blood、IQCK-Adipose、ATAD2B-Skin_fibroblast、UTP25-Adipose、ALG2-Adipose、LIN52-Liver
Rump width	COQ2-Adipose、PPP1R42-Intramuscular_fat、TTC22-Blood、ADIPOR2-Hypothalamus、FAM228A-Hypothalamus、UBXN2A-Blood、CSPP1-Blood、MOCS1-Adipose、PNRC2-Blood、SGK3-Adipose、WNT11-Macrophage
Rump angle	FAM228B-Liver、GTF3A-Embryo、ISCA2-Lung、MACROD1-Liver
Retained placenta	UNC13B-Blood、VPS35L-Liver、SLC31A1-Liver、SRM-Mammary、WNT11-Macrophage、SSTR2-Blood、FMNL2-Blood
Rear udder height	KCTD18-Adipose、SPATS2L-Blood、AOX1-Adipose、UNC13B-Blood、COQ2-Adipose、CHRND-Intramuscular_fat、SGK3-Adipose
Rear teat placemen	KCTD18-Adipose、SPATS2L-Blood、AOX1-Adipose、UNC13B-Blood、FAM228B-Liver、ALDH6A1-Liver、CHRND-Intramuscular_fat、ISCA2-Lung、PNRC2-Blood
Rear legs side view	STOML2-Mammary、ISCA2-Lung、MACROD1-Liver、SLC31A1-Liver、PROX2-Liver、RAB22A-Milk_cell、GK5-Liver
Rear legs rear view	UNC13B-Blood、STOML2-Mammary、TP53I3-Adipose、IFNLR1-Blood、IL22RA1-Rumen
Net merit	RPS6KA4-Uterus、TTC4-Blood
metritis	KCTD18-Adipose、SPATS2L-Blood、AOX1-Adipose、CHTF8-Liver、INMT-Liver
ketosis	KCTD18-Adipose、SPATS2L-Blood、TTC22-Blood、CHTF8-Liver、RPS6KA4-Uterus、TMOD2-Mammary、TRMT112-Lymph_node
hypocalcemia	KCTD18-Adipose、NCEH1-Blood、SSTR2-Blood、UTP25-Adipose、VAPB-Macrophage、AOX2-Mammary、ORC2-Liver
Front teat placement	KCTD18-Adipose、SPATS2L-Blood、AOX1-Adipose、CHRND-Intramuscular_fat、ISCA2-Lung、TSPEAR-Oviduct
Fore udder attachment	KCTD18-Adipose、SPATS2L-Blood、AOX1-Adipose、IQCK-Adipose
Feet and legs	COQ2-Adipose、STOML2-Mammary、TP53I3-Adipose、HPSE-Blood、IFNLR1-Blood、IL22RA1-Rumen
Displaced abomasum	PRG4-Liver
Dairy form	ALDH6A1-Liver、STOML2-Mammary、CHRND-Intramuscular_fat、CFAP410-Liver、ITGB2-Muscle、PROX2-Liver
Cow livability	STOML2-Mammary、PFKL-Intramuscular_fat、BSND-Uterus、FERMT3-Salivary_gland、HAS3-Blood
Body depth	KCTD18-Adipose、COQ2-Adipose、ALDH6A1-Liver、PPP1R42-Intramuscular_fat、TTC22-Blood、ADIPOR2-Hypothalamus、FAM228A-Hypothalamus、UBXN2A-Blood、CSPP1-Blood、SGK3-Adipose
Foot angle	SPATS2L-Blood、AOX1-Adipose、STOML2-Mammary、GTF3A-Embryo、NPEPL1-Macrophage、PLS1-Liver
Overall conformation score	KCTD18-Adipose、SPATS2L-Blood、AOX1-Adipose、UNC13B-Blood、COQ2-Adipose、KLHL29-Mammary

## Discussion

In this study, we aimed to investigate genomic evidence of selection signatures in Shanghai Holstein cattle using GGRS data. Two complementary approaches were applied for the detection of selection signatures in the studied population, i.e., the ROH and *iHS* methods, which should boost the accuracy of detection and eliminate unknown bias [26–28]. Overall, 101 and 18 candidate regions under selection were detected by the iHS approach and ROH estimation, respectively. These signatures provided insight into the genes contributing to the diverse phenotypes of these animals. Our results revealed a series of well-known and novel genes, such as *FAM135B, C2CD2, GOLGA4, ARFGEF1, CTDSPL, TSPEAR, SUPT3H, ATAD2B, KLHL29, FKBP2, STOML2* and *ECI2*, which are related to milk production; *IL22RA1, CALHM3, SNW1, PLXNA4, ABCA9, DDOST, ATP1B3, ALDH6A1* and *ADCK1*, which are associated with clinical mastitis; *SCS*, *ITGA9, FKBP1B, ACAT2, AMZ2, MRPL18, HDHD3, GNAS, VSX2, PLAC8, PXDC1, REG3G, DNAJB5* and *PRDX5*, which are involved in body temperature during heat stress; *NDUFB3, RGS3, UBD, DIS3L2, NRXN2, PEX14, SPTLC2, AQP1*, and *PTPN9*, which are involved in adaptation, especially climate adaptation, such as adaptation to tropical humidity and harsh environments; *SOD2, SNRPA1, TGFBR1, SLC39A11, PDE5A, HPSE* and *PRPF4B*, which are involved in reproduction events, *ST3GAL4, ALAD, NOD1* and *ITGB2*, which are involved in immune response, and *EXOSC10, MASP2* and *CSPP1*, which are candidate genes for longevity. To further understand the functional consequences of genetic variants on the cattle transcriptome, we explored the candidate genes on the cGTEx website.

According to the PCA based on genotypes, there was no obvious structure in the Shanghai Holstein cattle population. Kim et al. (2015) reported that modern dairy cattle populations are composed of both inbred (F ~ 0.1) and outbred structures because of the intensive use of a small number of influential males selected for artificial insemination (AI) and mated to cows that probably originated from common ancestors born more than three generations ago, which creates complex pedigree structures consisting of multiple inbreeding loops [17].

The SNPs used to detect regions associated with ROH were obtained from GGRS, a high-throughput reduced representation sequencing method used in our study. Similar to the genotyping-by-sequencing approach, this method has been designed for higher coverage (5×), which makes it more appropriate for outbred animals; thus, it is suitable for the Shanghai Holstein cattle population. However, the distribution of markers is usually uneven. In the past few years, several studies have explored the selection signatures of cattle, pigs, sheep and horses by next-generation sequencing. However, the detection of ROH is sensitive to the parameters or thresholds used for sequencing and pruning SNPs [29, 30]. The genotyping error in NGS data has an impact on ROH detection. Therefore, we allowed one heterozygous SNP per ROH to avoid losing particularly long ROH because of a single genotyping error; thus, the accuracy of detection in the NGS data was shown to be high after correction for bias by hidden errors in the genotyping data [21, 31]. For the impact of the density of SNPs, many studies have reported that more signatures of selection were able to be identified with NGS data than with SNP microarray data. NGS data facilitate the detection selection signatures at higher resolution than SNP array data. Moreover, NGS-based detection of ROH is more sensitive for short ROH that are typically missed using SNP array-derived genotypes [32]. In cattle, a low-density SNP microarray tends to overestimate the number of ROH that are shorter than 4 Mb, but using a dense chip leads to an underestimation of the number of long ROH ($\ge$8 Mb) [33, 34]. SNPs that are genotyped with low coverage can be used for detecting ROH. Compared with SNP array data, SNP genotyping with low coverage (4× on average) can achieve comparable detection of ROH by allowing a high number of heterozygous calls in the sliding window along the genome [29, 35].

In the past few years, many studies have been performed in the fields of horse, human and cattle genetics by the *iHS* method and the detection of ROH within populations [36–38]. Saravanan et al. (2021) implemented two complementary approaches viz. iHS and ROH to detect selection signatures for intra-population analyses in Indian cattle [28]. Pemberton et al. (2012) reported that the frequencies of ROH across the genome are correlated with signals of recent positive selection [39]. iHS is a measure of positive selection based on haplotype patterns. The positive correlation between the frequency of ROH and iHS was confirmed, supporting a role for natural selection in shaping genomic patterns of ROH. Regions with homozygosity in the long haplotypes were created by recent selection events. Short and intermediate ROH created by older selection events either have weaker iHS signals or are partially diluted by the presence of short and intermediate ROH generated by other forces. Zhang et al. (2015) reported that short ROH were selected and derived from ancient haplotypes that became fixed in populations, while long ROH were the result of more recent inbreeding events based on next-generation sequencing data in cattle populations [38]. Nolte et al. (2019) took genes falling into ROH islands and overlapping with iHS signals as input for enrichment analyses and found several pathways when the selection signatures were investigated in 942 stallions [36].

In this study, we focused mainly on detecting the footprints of selection left in the Shanghai Holstein cattle population after the process of introducing cattle from Canada, the USA, France and northern Europe. Despite their common relevance to Holstein breeding, the current breeding focus of these countries differs with respect to local climate conditions and national strategic directions. In addition, historically, the Shanghai Holstein cattle population underwent different breeding policies regarding pure and cross breeding and different primary foci of utilization. Shanghai Holstein has more than 100 years of history and is the offspring of Chinese native cattle and introduced breeds from Canada, the USA, France and northern Europe [40]. The Shanghai Holstein population is under high susceptibility to diseases such as mastitis, abortions and still births due to the harsh environment in Shanghai. Mastitis can decrease the yield and quality of milk in cattle. To reduce the environmental stress put on animals, exploring the mechanism of adaptation of livestock breeds to local climatic conditions for contemporary agriculture is important. Thus, the production of animals adapted to local climatic conditions can increase and be more environmentally friendly [41]. The method used here is an effective way to identify the genes relevant to important economic traits by identifying selection signatures within the genome. This method can also help us better understand the biological processes and mechanisms affected by ongoing strong artificial selection in livestock populations [31].

We found a total of 101 candidate regions, and 27 candidate regions representing strong signals (mean |iHS| score > 2) were identified using the iHS method. Fifty-nine significant regions containing 167 genes were located within the reproduction-related QTLs. Shanghai Holstein cows have only 2–3 parities over their entire life. The high proportion of candidate regions overlapping with reproduction-related QTLs indicated that this population was subjected to selection on reproductive traits, which was consistent with the fact that Shanghai Holstein has reproductive disorders [3]. Moreover, we found that 53 genes overlapped with candidate regions located within health-related or reproduction-related QTLs. Among these 53 genes, *SGO2, PPIL3, ORC2, NIF3L1, CLK1, BZW1, AOX1, AOX2, ALAD, BSPRY, CDC26, CFLAR, COX4I1, CTDSPL, EMC8, HDHD3, IRF8, ITGA9, MIR2367, MIR26A-1, MIR26C, NDUFB3, POLE3, PRPF4, RGS3, RNF183, SLC31A1, SLC31A2, VILL* and *C8H9orf43* overlapped with health-related QTLs. *ITGA9* is a heat shock response protein that is associated with body temperature during heat stress; it was discovered by conducting a genome-wide association study in a cattle population [42]. *COX4I1* has been shown to be associated with protein yield in Jersey cattle [43]. *NDUFB3* was identified to be involved in Mediterranean climate adaptations and morphology and stature [44], which supports the hypothesis that climate strongly influences body size because a smaller size is positively correlated with heat and aridity [45]. Taye et al. (2017) found that *ALAD* contributed to superior heat tolerance mechanisms in an African cattle population [46]. *ALAD* was also related to the inflammatory response [47]. *IRF8* is present within footprints of selection for Iraqi breeds and is linked to the acquired immune response to protozoan and bacterial infections [48]. Ben-Jemaa et al. (2020) provided an outline of potential selection signatures in North African cattle, and *IRF8* was found [49]. *RGS3* is an essential factor for the proper growth and development of calves [50]. Previous studies showed that *RGS3* was suggested to be a prognostic biomarker for small yellow follicle development in chickens. In rats, *RGS3* had negative regulatory functions in signalling. In humans, this gene was involved in GnRH responsiveness in granular cells [51]. In goats, *CTDSPL* was positively correlated with milk yield. Moreover, miR-26b and *CTDSPL* were significantly correlated with milk fat content [52]. *TGFBR1, SVIL, SNRPA1, SSTR2, SLC39A11, GMPS, CSPP1, COPS5, ARFGEF1* and *MYOM2* overlapped with reproduction-related QTLs, which overlapped with 8 candidate regions with mean |iHS| >2: BTA13:34,000,000–34,500,000, BTA14:30,750,000–31,250,000, BTA27:1,000,000–1,500,000, BTA21: 28,750,000–29,250,000, BTA13: 40,000,000–40,500,000, BTA8: 66,500,000–67,000,000, BTA1: 113,500,000–114,000,000, and BTA19: 59,750,000–60,250,000. Among these genes, *SVIL* overlapped with the candidate regions with the highest iHS score. This gene, which is a target of selection in seven Indian native cattle breeds, is associated with resistance to diseases/higher immunity [53]. *SNRPA1* is related to reproductive traits in livestock [54]. A comparison of selective regions and published QTL data suggests that *SLC39A11* is a candidate gene for reproductive traits. Association analysis demonstrates that *SLC39A11* has a substantial effect on the calving interval in dairy cattle [55]. *SLC39A11* was also shown to be associated with fore teat placement in the Chinese Holstein cattle population [56]. According to functional analysis, *SLC39A11* was a candidate gene for immunity and defence [57]. *CSPP1*, which was associated with reproduction, was detected by identifying the regions of signatures of selection across the genome of five Swedish breeds [31]. It is worth noting that the *CSPP1* gene has been described to play an important role in the productive life of Holstein cattle [58]. In the Shanghai Holstein cattle population, we observed quite high disease incidences for female fertility disorders. Many studies have found that there is a strong time-lagged effect of these female fertility disorders on longevity traits [59]. Genomic scans for selection signatures revealed that *ARFGEF1* is a hallmark gene for milk yield in cattle [28, 60].

In particular, 12 genes overlapping with 4 candidate regions related to heat tolerance QTLs, *ADCK1, GOLGA4, ITGA9, ECI2, PRPF4B, PXDC1, ADIPOR2, ALKBH1, DCP1B, SLIRP, SNW1* and *SPTLC2*, were detected in our study. Heat stress is an important problem compromising animal production and productivity in Shanghai, which is a subtropical region [61]. Heat stress induces heat shock, oxidative stress and osmotic stress, which are deleterious to normal cellular functions [62], such as reduced oocyte competence, thereby causing lower fertility [63]. Heat shock proteins play a crucial role in environmental stress adaptation and thermal balance [15]. *PRPF4B* and *PXDC1*, which are potential candidate genes affecting pregnancy establishment and maintenance in Chinese Holstein cattle, were identified by revealing the genetic and biological basis of the reproductive performance of dairy cows under heat stress [64–66]. The SNPs in *GOLGA4* explained the greatest proportion of the genetic variation in the cow conception rate in Holstein cows [67, 68]. *GOLGA4* was found to be associated with milk yield in many dairy populations [69]. *ITGA9* was identified in a large number of studies on the signature of selection and environmental adaptation to exposure to thermal stress in cattle [28, 70–72]. According to the study of Makina et al. (2015), *ADIPOR2*, which is related to reproductive performance, was identified by detecting signatures of selection within and between six cattle breeds in South Africa [73]. *DCP1B* was identified and considered a hub gene because it was involved in multiple pathways related to milk production [74]. SNW1 was considered to be a candidate gene associated with alternative subclinical mastitis traits [75].

We employed a strict criterion that the top 1 % of SNPs with the highest number of occurrences was chosen as an indication of a possible ROH island in the genome. The same threshold was reported in a study on cattle [25]. In our study, BTA21:31,435,280–33,563,201 was detected as a candidate region in the Shanghai Holstein cattle population. Yanhuang cattle are native Chinese cattle from South China. Small and medium ROH were found to be predominant in Yanhuang cattle, and three genes (*TMEM266, ETFA* and *ISL2*) at 32 Mb on BTA21 were also detected [76]. *ETFA* was explored on the cGTEx website and was reported to be significantly associated with 8 economically important traits, such as mastitis and overall conformation score. We detected candidate selection regions in the Shanghai Holstein cattle population by using ROH that harbour genes associated with milk production (*ABCA9, ALPL, C2CD2, CDC42*, and *PER2*), reproduction (*ABCC1, AIRN, CDX2, DCPS, ESPNL, ISL2*, and *MSX1*), resistance to diseases/higher immunity (*ABCC6, COMMD4, CSPG4, DDOST, KCNMB1, PLXNA4, RAP1GAP, SLIT3*, and *ST3GAL4*), heat stress (*ACAT2, AMZ2, GMPR2, MRPL18, PINK1, PKDCC, SOD2*, and *WTAP*), and feed efficiency or lipid metabolism (*AGPAT4, KIF17, NEIL1, PRKAR1A, SLC22A1*, and *THSD4*).

There are 11 genes detected by both methods. The *MTIF3* gene located on chromosome 12 has been found to inhibit the autophagy pathway in heat-shocked oocytes, affecting the mRNA abundance of *MTIF3* and further disrupting oocyte energy balance, this impairing embryonic development [77]. *UBD* has potential roles in innate immunity [78]. Zare et al. suggested *UBD* and *POLR1D* as candidates for susceptibility to *Mycobacterium avium subspecies paratuberculosis* infection [79]. A search for known QTLs affecting traits related to adaptation in the QTL database showed that *UBD* is located in the region between 15 and 40 Mb on chromosome 23 and harbours putative QTLs affecting cattle cell- and antibody-mediated immune response, tick resistance, heat tolerance, and respiratory rate [80]. *LNX2* was described earlier in the literature as a mastitis-related gene [81] and a candidate gene for residual body weight gain [82]. *USP12* plays an important role in stabilizing and enhancing the cellular function of androgen receptors, which are very important in regulating various sperm functions and have also been reported as fertility-related protein-coding genes in crossed cattle breeds [83].

To make use of meaningful annotations rather than simple annotations, we also conducted secondary GO term and KEGG pathway enrichment analyses, which have been proven to successfully identify overrepresented terms. Population-level analysis further suggests the presence of selection for adaptation in the Shanghai Holstein cattle population. A total of 413 genes embedded in selected regions belong predominantly to categories that are related to adaptation, such as molecular adaptation under heat stress. For example, ‘vitamin B6 metabolism’ (3 genes: *AOX1, AOX2* and *AOX4*; *P* value = 0.0045) is a growth- and development-related pathway that is under selection, as the metabolically active form of vitamin B6 aids in the synthesis of haemoglobin and enhances oxygen binding [84]. L-tryptophan (Trp) plays crucial roles in the balance between intestinal immune tolerance and gut microbiota maintenance. Tryptophan metabolism plays a pivotal role in human health [85]. Valine, leucine and isoleucine degradation and folate biosynthesis were enriched among genes harbouring SNPs associated with marbling score in Hanwoo cattle [86]. Abnormal folate metabolism has been causally linked with a myriad of diseases [87]. Purine metabolism (bta00230) was a unique KEGG pathway identified as significant in candidate regions for ROH. Purines and pyrimidines are vital constituents of DNA and RNA molecules as well as other substrates of lipid and carbohydrate metabolism. Additionally, purine metabolites are crucial for intracellular signalling and cellular energy, which can also act as cofactors to promote cell growth, proliferation and survival [88].

## Conclusions

Using two complementary analyses based on GGRS data, we constructed a high-resolution map of selection signatures in the Shanghai Holstein cattle population, which increases the spectrum of selective signals in the cattle genome. The selective signals identified in this study clearly reflected the stronger adaptation to hot and humid environments in Shanghai. Some candidate genes that might underlie differences in adaptation to specific environments and production systems were identified in potentially selected regions, such as *ITGA9, ACAT2*, and *PLAC8*. Genes associated with milk production, resistance to diseases, and reproduction were also identified as candidate regions in iHS and ROH analyses. We used cGTEx further to understand the functional consequences of genetic variants on the transcriptome of the Shanghai Holstein cattle population. Our findings may contribute to promoting the understanding of the genome evolution of and selection mechanisms in cattle.

## Materials and methods

### Genotypes

This study consisted of 1092 animals from the Shanghai Holstein population. The genotypes were sequenced using a genotyping by genome reducing and sequencing (GGRS) protocol (http://klab.sjtu.edu.cn/GGRS/) [89]. The data were extracted from our previous publication [90]. The raw reads with a base average quality score of at least 20 and a score of at least 30 in the first 65 bp aligned to the cow reference genome were retained. The filtered reads were aligned to the btau4.6 assembly of the cattle genome by using Burrows-Wheeler Aligner [91]. The SNP data were edited prior to statistical analyses. First, the SNPs that could not be mapped to the reference were removed. Afterwards, SNPs were excluded if they did not meet the following criteria: (1) calling quality greater than 20 (99 % accuracy); (2) sequencing depth on average greater than 5×; (3) call rate > 30 %; (4) minor allele frequencies (MAFs) ≥ 0.05; and (5) located on an autosome. Missing genotypes were imputed using iBLUP with the default parameters [92]. After quality control, the final data consisted of 164,312 autosomal SNPs. The mean distance between adjacent SNPs was 15.56 kb. Since the UMD3.1 assembly is used in most platforms, we converted SNP position information from btau4.6 to UMD3.1 using UCSC liftOver (https://genome.ucsc.edu/cgi-bin/hgLiftOver)[93], which is a tool for conversion between genome assemblies by coordinates.

### Population structure analysis

To evaluate the population structure of the Shanghai Holstein cattle population, principal component analysis (PCA) was conducted using GCTA (http://cnsgenomics.com/software/gcta/pca.html) [94]. The first three significant components were visualized using the R package “scatterplot3d”.

### Inbreeding coefficient estimations

The inbreeding coefficient was estimated through the -ibc command implemented in GCTA software, which returns three different inbreeding estimations, F_GRM_, F_HOM_ and F_UNI_. F_GRM_, calculated following VanRaden (2008), was equivalent to the estimate of an individual’s additive relationship to itself; F_HOM_, calculated following Wright (1948), was estimated based on the excess homozygosity; and F_UNI_ was calculated based on the correlation between uniting gametes, following Wright (1922).

### Detection of selection signatures using iHS

The integrated haplotype score (iHS) test was performed to investigate selection signatures in the Shanghai Holstein population. Before computing iHS, the ancestral allele of all bovine SNPs was inferred as the most common allele in the entire dataset, as described by Bahbahani et al. (2015) and Bertolini et al. ( 2018). The iHS score was calculated for each autosomal SNP using R’s “rehh” package [97], which uses a function to detect selection signatures in dense marker data using a test based on extended haplotype homozygosity (EHH) discussed above. The package was applied to estimate the statistics and generate plots to visualize and interpret the results.

The normal standardized iHS was calculated as

$$iHS=\frac{ln\left(\frac{{iHH}_{A}}{{iHH}_{D}}\right)-{E}_{p}\left[ln\left(\frac{{iHH}_{A}}{{iHH}_{D}}\right)\right]}{{SD}_{p}\left[ln\left(\frac{{iHH}_{A}}{{iHH}_{D}}\right)\right]}$$

where ${iHH}_{A}$ and ${iHH}_{D}$ represent the integrated EHH score for ancestral and derived core alleles, respectively. ${E}_{p}\left[ln\left(\frac{{iHH}_{A}}{{iHH}_{D}}\right)\right]$ and ${SD}_{p}\left[ln\left(\frac{{iHH}_{A}}{{iHH}_{D}}\right)\right]$ are the expectation and standard deviation in terms of frequency bin *p*. We divided the genome into 500-kb windows with a 250-kb overlap and used the averaged |iHS| value in each window as the test statistic. Windows at the top 1 % of the empirical distribution were considered to be candidate regions of positive selection.

### Runs of homozygosity analysis

We used the PLINK toolset version 1.9 to identify long series of consecutive, homozygous SNPs [98]. ROH were discovered using a sliding window of 100 SNPs, allowing one possible heterozygous genotype (to account for potential errors in genotyping and imputation) and 5 missing SNPs per window. The minimum SNP density was 1 SNP every 50 kb to ensure that low SNP density did not increase the length of the ROH, and the maximum distance between two consecutive homozygous SNPs in a run was kept at the default value of 100 kb. Homozygous genotypes with a length of > 1 Mb were defined as ROH. The algorithm in PLINK takes a window from a certain SNP and slides this window across the genome. Whether this window is a ROH was determined according to the previous criteria. The proportion of ROH windows encompassing each SNP was calculated. The percentage of animals that had the region with the most overlapping ROH on each chromosome was plotted. Statistical thresholds were determined empirically by selecting the top 1 % of the SNPs most commonly observed in ROH [37]. The number of ROH, the average and maximum length in kilobases and the number of SNPs were calculated for all the animals for chromosomes 1 to 29.

### Enrichment analyses of candidate genes under selection

Enrichment analysis is a promising strategy for increasing the likelihood of identifying biological processes that are highly related to the biological phenomena under study [99]. In this study, enrichment analyses for genes within each significant ROH island (top 1 %) and candidate region detected by the *iHS* method (the top 1 %) were performed. We used the database for annotation, visualization and integrated discovery (DAVID 6.8; https://david.ncifcrf.gov/summary.jsp) to extract biological features/meanings associated with the list of genes. Kyoto Encyclopedia of Genes and Genomes (KEGG) is a database resource for understanding the high-level functions and utilities of biological systems, such as cells, organisms and ecosystems, from molecular-level information, especially large-scale molecular datasets generated by genome sequencing and other high-throughput experimental technologies [100]. We explored the potential functions of the genes in all samples using KEGG; thus, enriched pathways with an adjusted *P* value < 0.05 were reported in our study.

### Aligning core regions to QTL database

QTLs were used to determine the expression patterns of these differentially expressed genes. Cattle QTLdb (http://www.animalgenome.org/cgi-bin/QTLdb/BT/index, updated Sep, 2014) contains 81,652 QTLs [101]. We used a Perl Script to conduct QTL-based annotation to identify all the significant candidate regions detected by the *iHS* method (top 1 %) contained or overlapped across the QTLs. The number and function of candidate regions was determined after annotation.

### cGTEx gene expression data set

Understanding the functional consequences of genetic variants on the transcriptome of livestock is essential for interpreting the molecular mechanisms underlying traits of economic value. In this study, to further investigate the hypothesis that many of our leading variants are regulatory, we retrieved multiple tissue gene expression data from cGTEx and then compared the gene expression data with a list of genes within each significant ROH island (top 1 %) and candidate region detected by the *iHS* method (the top 1 %). First, we retrieved the Ensembl IDs of the genes that were detected by ROH analysis and iHS simultaneously based on the annotation for cow genes (UMD3.1) available from the Ensembl database (http://www.ensembl.org/index.html). We uploaded the Ensembl IDs of the genes to cGTEx and downloaded all *cis-*eQTLs and *trans-*eQTLs related to genes from the website. Notably, because all the genome annotation files are based on ARS-UCD1.2 (https://www.ncbi.nlm.nih.gov/assembly/GCA_002263795.2; GenBank accession NKLS00000000.2) [102] for cGTEx, conversion of our SNPs located in the genes from UMD3.1 to ARS-UCD1.2 was performed using the UCSC liftOver tool [93]. Then, we used a Perl script to find SNPs shared between cGTEx and the SNP dataset in our study, thus identifying genetic variations that affect gene expression in specific tissues, which is a promising approach to finding functionally relevant pathways that contribute to traits.

TWAS integrates genome-wide association studies (GWAS) and gene expression datasets to identify gene-trait associations. We downloaded all 496 gene-tissue pairs significantly associated with 43 economically important traits from cGTEx. The TWAS results were compared with our genes that overlapped with candidate regions detected by iHS and ROH to detect common genes and to identify the genes that are significantly expressed in tissues relevant to a trait (P value < 0.05).

## Supplementary information

**Additional file 1**

**Additional file 2**

**Additional file 3**

**Additional file 4**

**Additional file 5**

## Data Availability

The SNP data are freely available at public repository Dryad (10.5061/dryad.cs133).

## Abbreviations

NGS: Next-generation Sequencing
SNP: Single Nucleotide Polymorphism
SCS: Somatic Cell Score
EHH: Extended Haplotype Homozygosity
iHS: integrated Haplotype Score
XPEHH: Cross-population Extended Haplotype Homozygosity
XP-CLR: Cross-population Composite Likelihood Ratio
ROH: Runs of Homozygosity
GO: Gene Ontology
KEGG: Kyoto Encyclopedia of Genes and Genomes
QTL: Quantitative Trait Loci
eQTLs: Expression Quantitative Trait Loci
cGTEx: Cattle Genotype-Tissue Expression atlas
GGRS: Genotyping by Genome Reducing and Sequencing
TWAS: Transcriptome-wide Association study
MAFs: Minor Allele Frequencies
AI: Artificial Insemination
