# Supplementary material for: Genome-wide selection signatures detection in Shanghai Holstein cattle population identified genes related to adaption, health and reproduction traits
Source: BMC Genomics. 2021 Oct 15;22:747. doi: 10.1186/s12864-021-08042-x (PMC8520274; doi:10.1186/s12864-021-08042-x)
Supplement: Supplementary file 1 — Additional file 1 [file 12864_2021_8042_MOESM1_ESM.docx]

**Table S1.** The mean distance between adjacent SNPs per chromosome.

| **Chr** | **Length (Mb)** | **No. of SNP** | **Mean Distance (kb)** | | **Standard Deviation** |
| --- | --- | --- | --- | --- | --- |
| chr1 | 158.34 | 7338 | 21.58 | 46.70 | |
| chr2 | 137.06 | 7049 | 19.44 | 42.21 | |
| chr3 | 121.43 | 8064 | 15.06 | 35.68 | |
| chr4 | 120.83 | 7572 | 15.96 | 39.06 | |
| chr5 | 121.19 | 7733 | 15.67 | 39.54 | |
| chr6 | 119.46 | 5312 | 22.49 | 45.49 | |
| chr7 | 112.64 | 7465 | 15.09 | 36.24 | |
| chr8 | 113.38 | 6088 | 18.62 | 39.76 | |
| chr9 | 105.71 | 5273 | 20.05 | 44.72 | |
| chr10 | 104.31 | 5952 | 17.53 | 36.65 | |
| chr11 | 107.31 | 7120 | 15.07 | 33.44 | |
| chr12 | 91.16 | 6640 | 13.73 | 35.26 | |
| chr13 | 84.24 | 6736 | 12.51 | 26.47 | |
| chr14 | 84.65 | 4004 | 21.14 | 42.58 | |
| chr15 | 85.3 | 5574 | 15.30 | 34.47 | |
| chr16 | 81.72 | 5269 | 15.51 | 35.24 | |
| chr17 | 75.16 | 4750 | 15.82 | 34.53 | |
| chr18 | 66 | 7579 | 8.71 | 22.01 | |
| chr19 | 64.06 | 6108 | 10.49 | 23.90 | |
| chr20 | 72.04 | 3395 | 21.22 | 46.00 | |
| chr21 | 71.6 | 5871 | 12.20 | 31.13 | |
| chr22 | 61.44 | 3765 | 16.32 | 31.90 | |
| chr23 | 52.53 | 4548 | 11.55 | 25.34 | |
| chr24 | 62.71 | 3662 | 17.12 | 35.49 | |
| chr25 | 42.9 | 5773 | 7.43 | 18.86 | |
| chr26 | 51.68 | 3536 | 14.62 | 34.27 | |
| chr27 | 45.41 | 3492 | 13.00 | 30.29 | |
| chr28 | 46.31 | 3680 | 12.58 | 29.73 | |
| chr29 | 51.51 | 5004 | 10.29 | 25.37 | |
| Total | 2512.08 | 164352 | 15.56 | 34.56 | |

**Figure S1.** Population structure from the principal component analysis. Population structure is shown as a 3D plot of the first three principal components (PC).


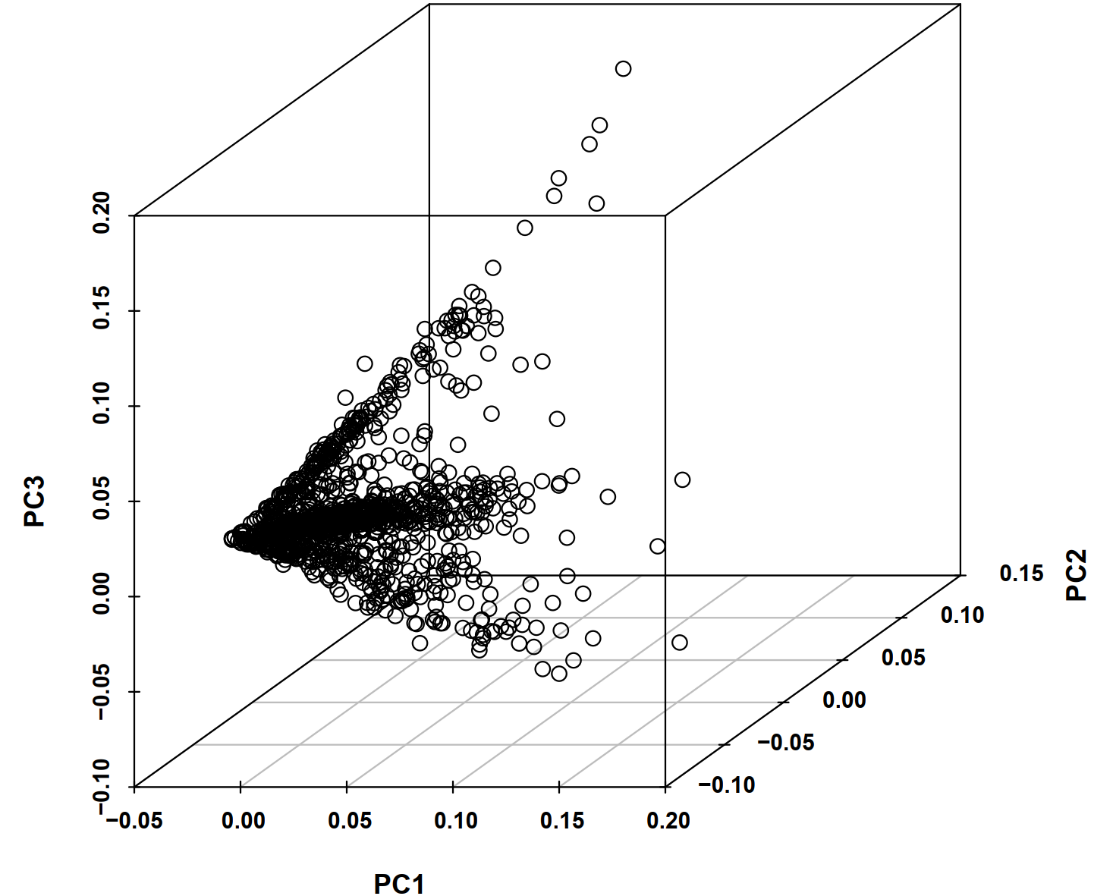


**Table S2.** Descriptive statistics of the inbreeding coefficient based on genomic information.

| **Inbreeding coefficient** | **Mean** | **Min** | **Max** | ***SD*** | ***N*** |
| --- | --- | --- | --- | --- | --- |
| F_GRM_ | 0.363 | 3.335 | 0.259 | 0.259 | 1092 |
| F_HOM_ | 0.363 | -2.699 | 0.884 | 0.488 | 1092 |
| F_UNI_ | 0.363 | -0.034 | 1.369 | 0.259 | 1092 |

**Table S3.** GO terms and KEGG pathways enriched (P value ＜ 0.05) based on genomic regions containing extreme |iHS| scores.

| **ID** | **Term** | **Count** | ***P* Value** |
| --- | --- | --- | --- |
| GO:0044424 | intracellular part | 158 | 4.82E-05 |
| GO:0071704 | organic substance metabolic process | 122 | 7.31E-05 |
| GO:0044237 | cellular metabolic process | 117 | 1.34E-04 |
| GO:0044238 | primary metabolic process | 115 | 2.51E-04 |
| GO:0005622 | intracellular | 160 | 3.95E-04 |
| GO:0006807 | nitrogen compound metabolic process | 81 | 9.63E-04 |
| GO:0043229 | intracellular organelle | 135 | 0.0024 |
| GO:0044710 | single-organism metabolic process | 51 | 0.0028 |
| KEGG_PATHWAY | Vitamin B6 metabolism | 3 | 0.0045 |
| GO:0051540 | metal cluster binding | 5 | 0.0062 |
| GO:0009056 | catabolic process | 29 | 0.0063 |
| GO:0043227 | membrane-bounded organelle | 133 | 0.012 |
| KEGG_PATHWAY | Folate biosynthesis | 3 | 0.012 |
| GO:0035821 | modification of morphology or physiology of other organism | 5 | 0.014 |
| GO:0048037 | cofactor binding | 9 | 0.015 |
| GO:0044422 | organelle part | 90 | 0.019 |
| GO:0044446 | intracellular organelle part | 88 | 0.021 |
| KEGG_PATHWAY | Tryptophan metabolism | 4 | 0.022 |
| KEGG_PATHWAY | Metabolic pathways | 25 | 0.027 |
| KEGG_PATHWAY | Valine, leucine and isoleucine degradation | 4 | 0.027 |
| GO:1990904 | ribonucleoprotein complex | 15 | 0.042 |
| GO:0043234 | protein complex | 54 | 0.049 |
| GO:0043902 | positive regulation of multi-organism process | 5 | 0.050 |

**Table S4.** GO terms and KEGG pathways enriched (P value ＜ 0.05) based on ROH islands.

| **ID** | **Term** | **Count** | ***P* Value** |
| --- | --- | --- | --- |
| GO:0044424 | intracellular part | 108 | 0.0020 |
| GO:0042995 | cell projection | 20 | 0.0047 |
| GO:0051091 | positive regulation of sequence-specific DNA binding transcription factor activity | 7 | 0.0048 |
| GO:0005622 | intracellular | 110 | 0.0053 |
| GO:0044464 | cell part | 125 | 0.0091 |
| GO:0044441 | ciliary part | 7 | 0.015 |
| GO:0016049 | cell growth | 8 | 0.017 |
| GO:0033036 | macromolecule localization | 28 | 0.018 |
| KEGG_PATHWAY | Purine metabolism | 6 | 0.018 |
| GO:0016740 | transferase activity | 27 | 0.024 |
| GO:0031090 | organelle membrane | 23 | 0.024 |
| GO:0048589 | developmental growth | 10 | 0.025 |
| GO:0097458 | neuron part | 13 | 0.039 |
| GO:0005929 | cilium | 8 | 0.040 |
| GO:0065009 | regulation of molecular function | 24 | 0.040 |
| GO:0004784 | superoxide dismutase activity | 2 | 0.044 |
| GO:0044238 | primary metabolic process | 79 | 0.049 |
